# Gear Classification in Skating Cross-Country Skiing Using Inertial Sensors and Deep Learning

**DOI:** 10.3390/s24196422

**Published:** 2024-10-04

**Authors:** Antonio Pousibet-Garrido, Aurora Polo-Rodríguez, Juan Antonio Moreno-Pérez, Isidoro Ruiz-García, Pablo Escobedo, Nuria López-Ruiz, Noel Marcen-Cinca, Javier Medina-Quero, Miguel Ángel Carvajal

**Affiliations:** 1 ECsens, Department of Electronics and Computer Technology, Sport and Health University Research Institute (iMUDS-UGR), Research Centre for Information and Communications Technologies (CITIC-UGR), University of Granada, 18071 Granada, Spain; antoniopoug@ugr.es (A.P.-G.); juanantoniomp@ugr.es (J.A.M.-P.); isirg@ugr.es (I.R.-G.); pabloescobedo@ugr.es (P.E.); nurilr@ugr.es (N.L.-R.); 2 Department of Computer Engineering, Automatics and Robotics, Research Centre for Information and Communications Technologies (CITIC-UGR), University of Granada, 18071 Granada, Spain; auro@ugr.es (A.P.-R.); javiermq@ugr.es (J.M.-Q.); 3 Department of Health Sciences, University of San Jorge, Villanueva de Gállego, 50003 Zaragoza, Spain; nmarcen@usj.es

**Keywords:** inertial measurement unit (IMU), deep learning, cross-country skiing, embedded system

## Abstract

The aim of this current work is to identify three different gears of cross-country skiing utilizing embedded inertial measurement units and a suitable deep learning model. The cross-country style studied was the skating style during the uphill, which involved three different gears: symmetric gear pushing with poles on both sides (G3) and two asymmetric gears pushing with poles on the right side (G2R) or to the left side (G2L). To monitor the technique, inertial measurement units (IMUs) were affixed to the skis, recording acceleration and Euler angle data during the uphill tests performed by two experienced skiers using the gears under study. The initiation and termination points of the tests were controlled via Bluetooth by a smartphone using a custom application developed with Android Studio. Data were collected on the smartphone and stored on the SD memory cards included in each IMU. Convolutional neural networks combined with long short-term memory were utilized to classify and extract spatio-temporal features. The performance of the model in cross-user evaluations demonstrated an overall accuracy of 90%, and it achieved an accuracy of 98% in the cross-scene evaluations for individual users. These results indicate a promising performance of the developed system in distinguishing between different ski gears within skating styles, providing a valuable tool to enhance ski training and analysis.

## 1. Introduction

The advancements in electronics and inertial sensors offer novel possibilities for monitoring human activities using compact and lightweight systems [1,2,3,4]. This sensorization enables the collection of valid movement data without affecting the corresponding biomechanical features in a complex sport with the objective of improving professional techniques and preventing injuries [5,6]. The advances in this type of analysis are even more significant in sports like cross-country skiing, which are performed in challenging environments such as ski tracks [7]. This precise analysis allows for a detailed examination of the technique in such demanding conditions.

In this way, different ski disciplines have been studied using inertial or positional sensors, such as alpine skiing [8] or cross-country skiing (XC), both classic [9,10] and skating styles [11]. Both XC skiing techniques involve multiple gears. A gear is defined as a movement pattern characterized by specific coordination between upper and lower body movements [12,13]. Different gears are used in both styles, depending on factors such as slope, skier fitness and technique, snow conditions, and speed of movement, among others. In the skating technique, four primary gears have been identified, ranging from gear-1 (G1) to gear-4 (G4). G1 is a recreational gear that is not typically used in competitive racing. G2 is an uphill technique for ascending steep slopes at low speeds, involving skis diverged at a wider angle than other techniques and asymmetric pole planting every two ski pushes. Therefore, two sub-techniques can be distinguished by direction: on the right (G2R) or at the left side (G2L). For less steep inclines or higher fitness levels, a different gear, G3, is used. G3 is a symmetric technique characterized by a cycle featuring a parallel pole plant with each lower-body skating stroke [14]. Finally, G4 is a technique used in flat terrain, with less divergence of skis and pole planting occurring every two ski strokes. Consequently, the divergence of skis, angulation, and force momentum vary between each technique or sub-technique [15].

During training sessions or competitions, skiers continuously switch between techniques to adapt to the ski track and conditions. Therefore, analyzing the timing and track sections where each gear is used is highly valuable for athletes and coaches seeking to improve skier performance. Typically, coaches cannot observe skiers throughout the ski track due to rugged terrain or vegetation that blocks visibility. Analysis of uphill techniques is particularly important since, as suggested by Torvik et al. (2019), 50% of race time and most time differences during a race occur in uphill sections [14]. Therefore, it would be especially valuable to have a system capable of analyzing the duration during which a skier uses each technique to establish a correlation between the different sections of the ski track and the corresponding technique used.

In this context, the objective of the present work is to develop a system capable of distinguishing three distinct styles of cross-country skiing by using inertial measurement units directly embedded in the skis in conjunction with a suitable deep learning model [16,17,18]. In this study, convolutional neural networks combined with long- and short-term memory are proposed to classify and extract spatio-temporal features.

## 2. Related Works

In this section, previous studies related to the current research are summarized, with special attention given to the number of sensors used and the accuracy achieved for gear classification by different authors.

Stöggl et al. [15] analyzed the gear used in the skating style using a smartphone placed on the athlete’s chest to monitor the acceleration on rollers on a treadmill. A machine learning model was trained with the collected data, achieving a technique identification accuracy of 86.0 ± 8.9% using the collective data and 90.3 ± 4.1% (*p* < 0.01) when the data were separated per individual skier. Similarly, Rindal et al. developed algorithms for classifying sub-techniques in skiing, achieving a high accuracy of 93.9% [19]. However, placing a smartphone on the chest may be uncomfortable for some athletes and would be unsuitable for racing environments. Gløersen et al. further expanded on this work, using carrier-phase differential global navigation satellite system (dGNSS)-based tracking to classify skating sub-techniques and cycle characteristics in cross-country ski skating [20]. This was achieved using a neural network classifier on position measurements from a head-mounted dGNSS antenna.

A solution based on wearable sensors directly fixed to the skis could be more practical in real-world situations [21]. With the advancements in wearable sensor technology, movement can be monitored in a less invasive manner. In this field, Skisens AB provides an analysis of the power applied to the poles using gauge-based sensors and inertial measurement units (IMUs) integrated into the handle, which transmit data to a smartphone for power calculation.

During the monitoring of training or competition sessions, a large amount of data is collected, making artificial intelligent models particularly suitable for managing and processing these data [22]. Johansson et al. [11] processed the data reported by two skiers and the Skisens AB system using machine learning to identify the techniques in the skating style. An accuracy of 95% was achieved using the model with the two same skiers. However, this precision fell to 78% when the model was applied to a third skier. In that study, all tests were carried out with rollers in varied terrain and conditions. Jang et al. [23] studied different configurations of gyroscope sensors placed on skiers’ bodies to monitor classical and skating styles on outdoor flat and natural courses. A machine learning model trained with data from three skiers achieved a maximum accuracy of 90% for the skating style using seventeen IMUs, which dropped to 85% when the number of sensors was reduced to five inertial sensors. Sakurai et al. [24] reduced the number of inertial sensors to four IMUs to study the classification of three gears in skating style on rollers on asphaltic outdoor roads. Two IMUs were placed on the wrists, and the other two in front of the roller ski bindings. Data were processed with an automated system based on a decision tree, achieving an overall accuracy of 95%. The system was developed using data from one skier and validated with eight male and seven female college cross-country skiers.

**Figure 1:**
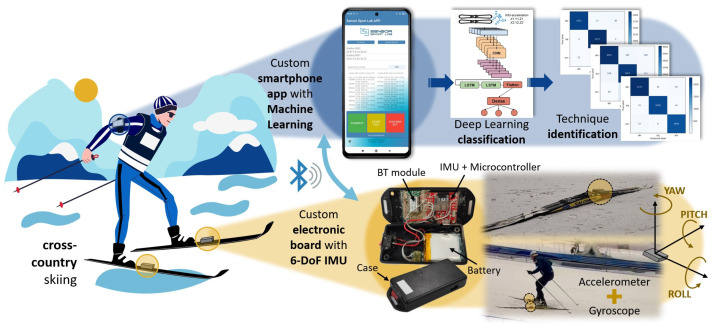
System overview of gear classification in skating cross-country skiing using inertial sensors and deep learning.

The aim of this current work is to identify the different uphill skating gears using the minimum number of inertial sensors and an artificial intelligence model to process the data. The two IMUs used were placed directly on each ski, making the entire system portable and suitable for testing in real training and competition conditions while also ensuring comfort for the skiers (see Figure 1).

## 3. Materials and Methods

### 3.1. Materials

The tests were carried out using a modified version of our previously developed system [4]. The customized electronic board integrates an MPU-9250 (Invensense, San Jose, CA, USA) 9-degree-of-freedom IMU, which encompasses a 3-dimensional accelerometer, gyroscope, and magnetometer. In addition, the system incorporates an onboard SAMD21 low-power microcontroller (Microchip Technologies Inc., Chandler, AZ, USA).

The inertial motion sensor comprises a TDK-INVN 6-axis IMU MPU-6500 with a configurable acceleration range from ±2 g to ±16 g and a configurable gyroscope range from ±250 to ±2000 degrees per second (dps). It also features an AKM-8963 magnetometer (AKM Semiconductor, Inc., Tokyo, Japan) with a range of 4800 µT connected through the AUX-I2C port of the MPU-6500. This inertial measurement unit includes digital motion processor (DMP) technology, capable of filter processing and accurate calculation of three-dimensional orientation, or the so-called attitude heading reference system (AHRS), to determine the inertial angles of yaw, roll, and pitch.

**Figure 2:**
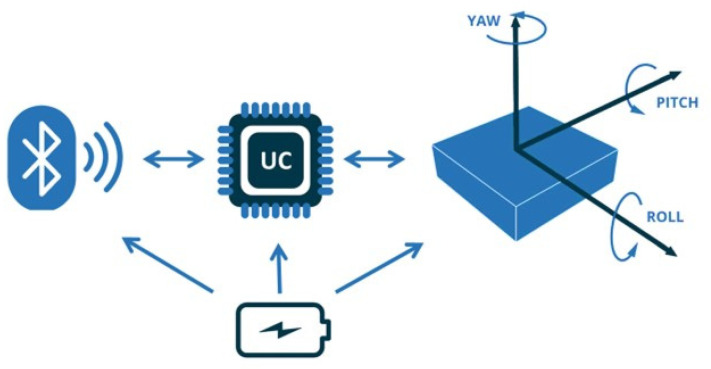
General block diagram of the system, illustrating the interconnection between the main components. The microcontroller unit (µC) receives data from the IMU and connects to the Bluetooth module for transmission to the end user. A battery supplies power to all system components.

For three-dimensional orientation, sensor data fusion could be performed in the DMP to obtain rotation quaternions, which can then be used to calculate the Euler angles (pitch, roll, and yaw). However, in the present work, only the accelerometer data are used to minimize the sensor system’s complexity. Consequently, the three components of the acceleration are transmitted to the user’s smartphone via Bluetooth and are also saved on an onboard SD as a backup. Both the SD card and the BM78 Bluetooth module (Microchip Technologies Inc., Chandler, AZ, USA) are integral components of the custom-developed electronic board. Figure 2 shows the block diagram of the electronic instrumentation.

To power the system, a 450 mAh battery is used, allowing continuous operation for over ten hours. Detailed consumption information is described in Table 1. It should be mentioned that a sample rate of 112 Hz was selected, and the accelerometer and gyroscope were configured in their maximum ranges (±16 g and ±2000 dps, respectively). The IMU incorporates an internal temperature sensor that is used to monitor the system temperature. This feature is crucial because the IMU operates under low-temperature conditions, and the well-being of the battery could be influenced by these conditions.

### 3.2. Experimental Setup

**Figure 3:**
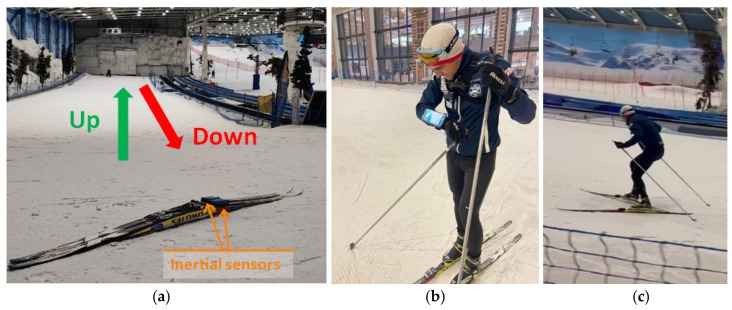
Experimental setup for dataset collection: (**a**) The ski track slope where the dataset was collected and inertial sensors placed on the skis. (**b**) One of the skiers uses the smartphone application to control the dataset collection, and (**c**) skier applying G3 during one climbing.

**Figure 4:**
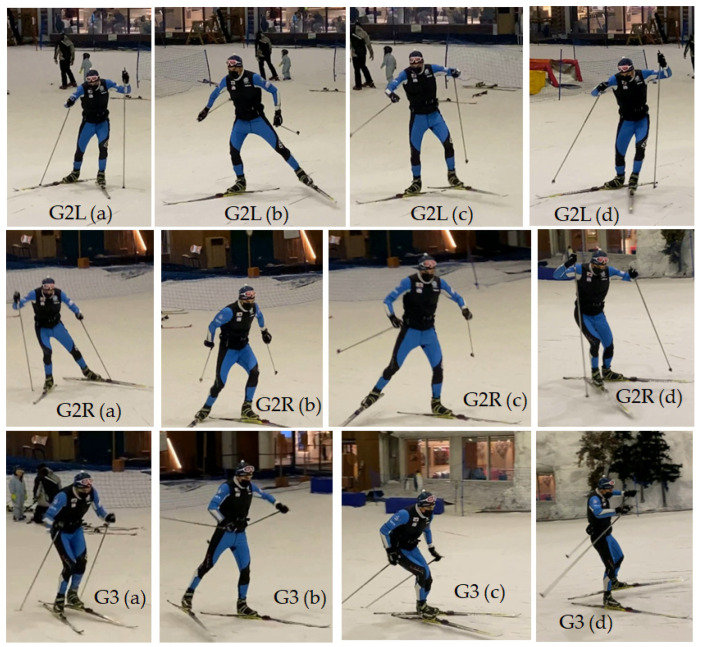
Illustration of cross-country skiing skating gears. G2L (**a**) planting poles while gliding with the left ski; (**b**) gliding with the right ski; (**c**) recovering arms; (**d**) planting poles at the end of the cycle. G2R (**a**–**d**) is similar to G2L but with a push-off using the right ski while gliding. G3 (**a**) plating poles while gliding with the right ski; (**b**) recovering arms; (**c**) planting poles while gliding with the left ski; (**d**) recovery and end of cycle.

**Figure 5:**
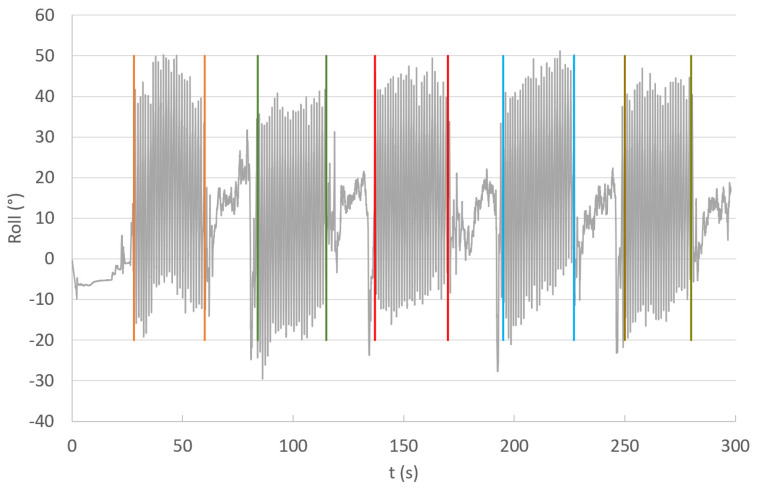
The roll angle of the left ski was recorded during five consecutive series using G2L. The uphills are marked between vertical bars with different colors.

The dataset comprises experimental data recorded during multiple climbs at the SnoZone indoor ski resort located in Arroyomolinos (Madrid, Spain), as shown in Figure 3. IMUs could be attached to any ski, allowing their use in real conditions with any skier without affecting their technique. The inertial parameters of two subjects were recorded during uphill climbs using the three different gears under study, depicted in Figure 4. National-level skiers aged 45 and 40 climbed a 100 m uphill with a moderate slope of 10%. Each skier completed 20 climbs using each analyzed uphill technique: 20xG2R, 20xG2L, and 20xG3. The IMUs were covered with plastic film to waterproof them and fixed to a pair of Salomon SK10 skis using double-sided tape, as shown in Figure 3. Five series of four climbs per technique were recorded, recalibrating the IMUs at the beginning of each set of climbs to ensure accurate monitoring without exceeding the recommended usage time for high-accuracy mode monitoring, as reported in previous work [4]. Figure 4 displays frames from the data collection process for the three gears under study, illustrating the differences between them. The data collected per sequence were analyzed and segmented to discard downhill sections, as illustrated in Figure 5.

### 3.3. Smartphone Application

A smartphone application was developed with the primary purpose of controlling the designed hardware and data acquisition, allowing simultaneous connection to two different devices, the IMUs for this study, and allowing synchronized measurements. The connection was established via Bluetooth, eliminating the need for additional cables.

For synchronization of both systems, the connection process is initiated by sequentially establishing a Bluetooth link with each device. Once that is established, the devices are ready to communicate with the application. The control is carried out by sending different commands that initiate or stop the measurements to achieve the desired synchronization. Data are stored on the SD card during the acquisition process. In addition, the smartphone stores the data in a backup file. After each test, the data are downloaded from the SD memory for subsequent processing and analysis.

**Figure 6:**
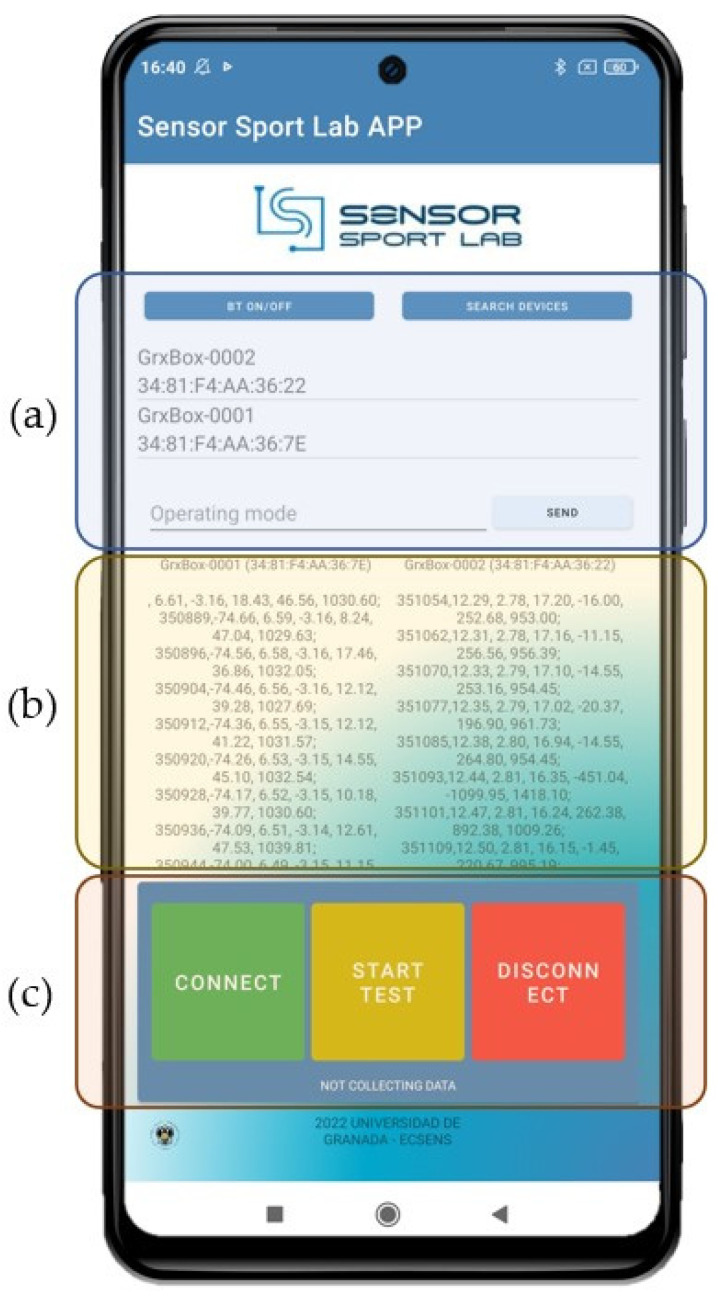
Screenshot of the control application for dataset collection: (**a**) Devices detected and connected to the smartphone via Bluetooth protocol. (**b**) Data collected from the two inertial systems. (**c**) Control buttons for connecting devices and starting/stopping data collection.

In terms of user interface, a key design requirement was simplicity, thus providing ease of use in training environments and situations where user-friendly operation and rapidity of acquisition are crucial. As shown in Figure 6, the designed user interface can be divided into several parts: (a) Bluetooth connection interface: This enables the search for nearby Bluetooth devices, displaying only the hardware developed after filtering. Additionally, it features a bar for free control of the devices, allowing the sending of any command. (b) Communication panel: This allows visualization of both the names of the connected devices and the real-time data received from each. (c) Quick communication panel: comprising three buttons for rapid control of tests. The green button establishes a link with the devices, the yellow button sends the measurement start command once connected, and the red button terminates the connection.

The application was programmed on a Xiaomi RedMi Note 9 using Android Studio IDE (Arctic Fox, version 2020.3.1) and is compatible with Android version 12.0 (API level 31).

### 3.4. Deep Learning Classification Model

Initially, the raw data collected by the inertial measurement units undergoes a segmentation process. A data stream S from a sensor s consists of a sequence of measurements $s=\left\{s_{0},\ldots ,s_{i}\right\}$. Each measurement $s_{i}$ is related to a different timestamp $t_{i}$ within the specified interval $\left[t_{0},t_{N}\right]$. The study utilizes input sensors that capture 3-axis acceleration (X, Y, Z) from an IMU-based device on each ski board. Due to the device’s high sampling rate, a segmentation technique was developed to aggregate and resample the raw data into a streamlined and uniform stream. This process involves the short-term collection of samples within temporal windows, which have been proven to effectively represent sports activities [1,3,25]. The median function is used to compute the aggregation function.

Furthermore, a time step parameter Δ = 250 ms is introduced to accurately identify specific times to collect a data sequence and label it according to the gear performed. Real-time models require the use of both current and previous measurements; therefore, for each timestamp $t_{i}$, a sequence W is defined, consisting of prior data $S_{i}^{*}$, spanning 320 samples (equivalent to 2.5 s), and noted as W = 320. Therefore, at least 3 s should be processed as the sample for gear classification. This sequence is maintained for each axis (X1, X2, Y1, Y2, Z1, Z2) of the IMU sensors on both ski boards (the IMU called GrxBox number 1 was placed on the left ski and number two on the right ski, see Figure 6a). The size of the sequence is consistent across all sources $S_{s}$ and is organized as follows:

(1) $$S_{i}^{*}=S_{i}\to S_{\left\{i-1\right\}},\ldots ,S_{\left\{i-W\right\}}$$

Based on the sequence of sample data, sized *Wx6*, the predictions are formulated according to the activity labeled by the ski user. Three gears have been identified: G2R, G2L, and G3. The association between the acceleration measured by the IMU devices on the ski board and the gear is determined through a classification model. A deep learning model employing CNN and LSTM architecture is suggested for this task. This state-of-the-art architecture has demonstrated its superiority in our previous research [1,3], as well as in the research of other authors [26,27,28] in the domains of IoT and sensor-based predictions.

**Figure 7:**
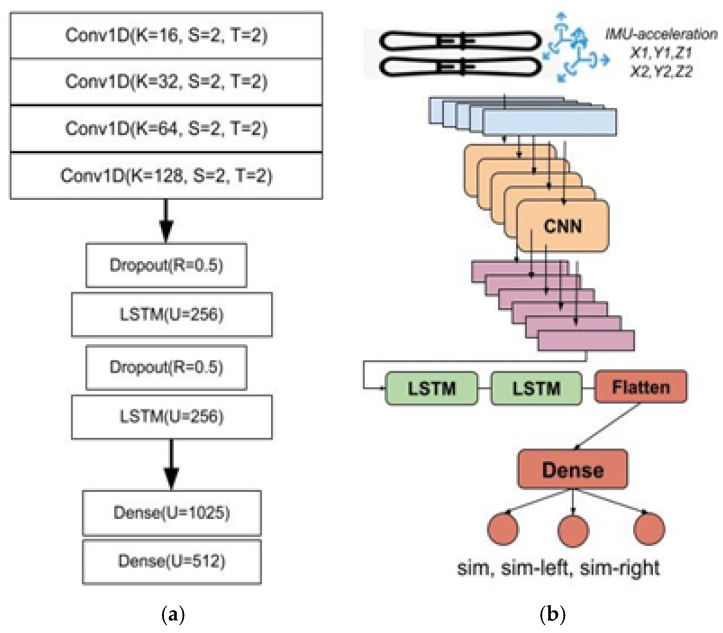
CNN-LSTM model for the classification and estimation of ski exercises based on the acceleration data from ski boards. (**a**) Detailed description of the layers and parameters (K = number of convolutional filters; S = kernel size; T = stride size of the filter; R = dropout rate; U = number of units in the LSTM and dense layers). (**b**) A conceptual overview of the architecture illustrating Conv1D layers for local feature extraction and LSTMs for capturing temporal dependencies in the sequential data.

Initially, three 1D CNN layers, termed Conv1D(K,S,ST), where K is the number of kernels, S is the convolution size, and ST is the stride, are used as spatial feature extractors with a stride of 2 to halve the dimension of the input matrix in the output matrix, and include a ReLU activation function to address vanishing gradients and enhance training efficiency. The temporal patterns of the spatial features extracted by CNN are modeled using a two-layer LSTM network with 32 units each. Subsequently, two dense layers based on multilayer perceptrons map the spatio-temporal features to the final output, which consists of three units corresponding to the classes (Sym (G3), asym left (G2L), and asym right (G2R)), utilizing categorical cross-entropy as the loss function with the Adam optimizer, and measuring performance through accuracy. In Figure 7, the architecture and layers of the CNN-LSTM model are detailed.

## 4. Results

### Deep Learning Model Performance

This section presents the case study conducted to evaluate the performance of our ski-gear prediction model. The model is designed to classify ski-training exercises performed by a user into three distinct gears: G2L, G2R, and G3. We conducted a case study by recording a dataset in which two users performed five ascents in each gear over two scenes, resulting in a total of 12,390 recorded instances of the different gears described above. This dataset comprised 4350 instances of G2R, 4217 of G2L, and 3823 of G3. As mentioned in the previous section, the data used for both training and model evaluation were derived from the accelerometers (axes X1, X2, Y1, Y2, Z1, and Z2) of the sensors attached to each ski board, left and right. To thoroughly assess the data, we adopted different evaluation settings:

- Intra-User Evaluation with Crossed Scenes: Each user’s data was split into two scenes, with one scene (comprising five ascents per gear) used for training and the other for evaluation. This process was reversed in a second iteration to ensure robustness in the evaluation. This method aimed to assess the model’s ability to learn and predict the ski gears for the same user under varying conditions. Table 2 and Table 3 present the precision, recall, F1 score, and support metrics for each class (G2R, G2L, and G3) for user 1 and user 2, respectively. Overall accuracy, macro-average, and weighted average values are also provided, demonstrating the model’s effective performance in classifying skating gears for individual users.

**Figure 8:**
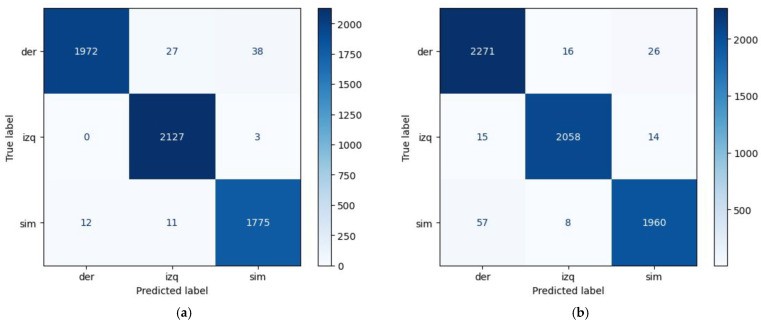
Confusion matrices for intra-user evaluation. (**a**) ‘User 1’ with a total of 5965 samples. (**b**) ‘User 2’ with a total of 6425 samples.

The performance of the model, when trained and tested on user 1’s data, demonstrated an accuracy of 98%. The precision, recall, and F1 scores were consistently high for the symmetric gears (G3), the asymmetric right gear (G2R), and the asymmetric left gear (G2L), with a perfect recall achieved for the G2L gear. The confusion matrix demonstrated minimal misclassification between gears (see Figure 8). In the case of user 2, the model also exhibited high precision, achieving an overall rate of 98%. The precision, recall, and F1 scores across all gears indicated strong performance, particularly for G3 and the asymmetric gears (G2L and G2R), which showed excellent recall.

- Cross-user evaluation: The model was trained using data from user 1 and evaluated using data from user 2 and vice versa. This approach allowed us to assess the model’s generalizability between different users. Table 4 presents the precision, recall, F1 score, and support metrics for each class (G2R, G2L, and G3) in the cross-user evaluation.

**Figure 9:**
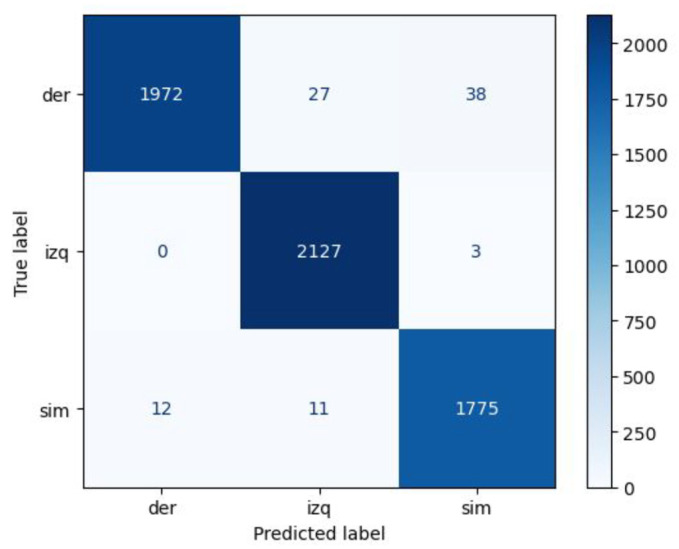
Confusion matrix for cross-user evaluation.

In a highly demanding evaluation scenario with cross-user validation (training from user 1 and testing on user 2, and vice versa), the model achieved an overall precision of 90%. Although the precision and recall rates for the right asymmetric gear remained robust, the left asymmetric gear exhibited some difficulties, resulting in a lower recall and F1 score, as shown in the confusion matrix (see Figure 9).

These findings highlight the model’s ability to accurately classify skiing gears, with particularly strong performance in distinguishing between the G2R, G2L, and G3 gears. The performance drop of the model in the cross-user evaluation points to potential challenges in generalizing the model across different users, indicating an area for further investigation and model refinement. The dataset and the models are available in the following repository: https://github.com/AuroraPR/Skiing-Classification (accessed on 1 October 2024), which includes the implementation of the proposed methods with Python 3.9.13 and Keras 2.11.0.

To highlight the main novelties and improvements reached by the system presented in the current work, the performance of the different classification systems described above has been summarized in Table 5, which provides a detailed overview of related works.

To evaluate the improvement of the presented system, it should be considered that using only the data reported by two 3D accelerometers and the machine learning model described above, the accuracy obtained was higher or similar to other more complex systems, as shown in Table 4.

In fact, the overall accuracy was 98% in the case where the training was carried out with the same skier (no cross-training) and 90% for cross-training with two skiers. Therefore, the system presented is simpler and more comfortable to use than others, making it suitable for field tests in real training sessions and reporting with high accuracy.

## 5. Conclusions

This work demonstrates the viability of detecting and classifying different gears of the skating style in cross-country skiing using two inertial measurement units placed on the skis along with a machine learning model. Acceleration values were sufficient to discriminate between different gears (G2R, G2L, and G3) during uphill, achieving an accuracy of 98% for individual skier training processes and dropping slightly to an acceptable 90% when using cross-skier data for training. The performance of the proposed system validates the inertial sensor system, the smartphone application, and the deep learning model described in the current work for their application to gear classification in cross-country skiing in the skating style, opening the possibility of application to other skiing styles.

## Figures and Tables

**Table 1:** Consumption specifications for the developed system.

Operation Mode	Consumption (mAh)	Battery Life (h)
Not connected	25.60	17.60
Connected	30.10	14.95
Transmitting data	39.30	11.45
Sleep mode	0.35	1285.70 (53.57 days)

**Table 2:** Intra-user evaluation metrics for user 1.

Class	Precision	Recall	F1 Score	Support
G2R	0.99	0.97	0.98	2037
G2L	0.98	1.00	0.99	2130
G3	0.98	0.99	0.98	1798
Accuracy	0.98			
Macro avg	0.98			
Weighted avg	0.98			

**Table 3:** Intra-user evaluation metrics for user 2.

Class	Precision	Recall	F1 Score	Support
G2R	0.97	0.98	0.98	2313
G2L	0.99	0.99	0.99	2087
G3	0.98	0.97	0.97	2025
Accuracy	0.98			
Macro avg	0.98			
Weighted avg	0.98			

**Table 4:** Cross-user evaluation metrics.

Class	Precision	Recall	F1 Score	Support
G2R	0.94	0.93	0.93	4350
G2L	0.84	0.92	0.88	4217
G3	0.94	0.84	0.88	3823
Accuracy	0.90			
Macro avg	0.90			
Weighted avg	0.90			

**Table 5:** Related works comparison table.

	Stöggl et al. [15]	Johansson et al. [11]	Jang et al. [23]	Sakurai et al. [24]	Our System
Number of gears	5	3	4	6	3
Sensor system used	IMU and smartphone GPS	Power meters: Force sensors and IMU	Gyro	6-DoF IMUs	3D accelerometer
Number of sensors	1 + 1	2 + 2	17	4	2
Data processing	Markov chain of multivariate Gaussian distributions	CNN, BLSTM, and LSTM architecture	CNN-LSTM architecture	Decision tree	CNN and LSTM architecture
Accuracy	90%	95%	90%	-	98%
Cross-user accuracy	-	78%	-	95%	90%

## Data Availability

The data presented in this study are available at https://github.com/AuroraPR/Skiing-Classification (accessed on 1 October 2024).
